# Introducing the DizzyQuest: an app-based diary for vestibular disorders

**DOI:** 10.1007/s00415-020-10092-2

**Published:** 2020-07-25

**Authors:** E. C. Martin, C. Leue, P. Delespaul, F. Peeters, A. M. L. Janssen, R. Lousberg, A. Erdkamp, S. van de Weijer, J. Widdershoven, H. Blom, T. Bruintjes, A. Zwergal, E. Grill, N. Guinand, A. Perez-Fornos, M. R. van de Berg, J. J. A. Stultiens, H. Kingma, R. van de Berg

**Affiliations:** 1 Division of Balance Disorders, Department of Otorhinolaryngology and Head and Neck Surgery, Maastricht University Medical Center+, Maastricht, The Netherlands; 2 Department of Psychiatry and Neuropsychology, School for Mental Health and Neuroscience, Maastricht University Medical Center+, Maastricht, The Netherlands; 3 Department of Clinical Psychological Science, Faculty of Psychology and Neuroscience, Maastricht University, Maastricht, The Netherlands; 4 Department of ENT/Audiology, School for Mental Health and Neuroscience (MHENS), Maastricht University Medical Centre, Maastricht, The Netherlands; 5 Department of Methodology and Statistics, Care and Public Health Research Institute (CAPHRI), Maastricht University, Maastricht, The Netherlands; 6 Department of Psychiatry and Psychology, Maastricht University Medical Center, Maastricht, The Netherlands; 7 Department of ENT, HagaZiekenhuis, The Hague, The Netherlands; 8 Apeldoorns duizeligheidscentrum, Gelre ziekenhuizen, Apeldoorn, The Netherlands; 9 Department of Neurology, Ludwig-Maximilians-University of Munich, Munich, Germany; 10 Department of Medical Informatics, Ludwig-Maximilians-University of Munich, Munich, Germany; 11 Service of Otorhinolaryngology Head and Neck Surgery, Department of Clinical Neurosciences, Geneva University Hospitals, Geneva, Switzerland; 12 Faculty of Physics, Tomsk State Research University, Tomsk, Russia

**Keywords:** DizzyQuest, Experience sampling, Vestibular disorders

## Abstract

**Background:**

Most questionnaires currently used for assessing symptomatology of vestibular disorders are retrospective, inducing recall bias and lowering ecological validity. An app-based diary, administered multiple times in daily life, could increase the accuracy and ecological validity of symptom measurement. The objective of this study was to introduce a new experience sampling method (ESM) based vestibular diary app (DizzyQuest), evaluate response rates, and to provide examples of DizzyQuest outcome measures which can be used in future research.

**Methods:**

Sixty-three patients diagnosed with a vestibular disorder were included. The DizzyQuest consisted of four questionnaires. The morning- and evening-questionnaires were administered once each day, the within-day-questionnaire 10 times a day using a semi-random time schedule, and the attack questionnaire could be completed after the occurrence of a vertigo or dizziness attack. Data were collected for 4 weeks. Response rates and loss-to-follow-up were determined. Reported symptoms in the within-day-questionnaire were compared within and between patients and subgroups of patients with different vestibular disorders.

**Results:**

Fifty-one patients completed the study period. Average response rates were significantly higher than the desired response rate of > 50% (*p* < 0.001). The attack-questionnaire was used 159 times. A variety of neuro-otological symptoms and different disease profiles were demonstrated between patients and subgroups of patients with different vestibular disorders.

**Conclusion:**

The DizzyQuest is able to capture vestibular symptoms within their psychosocial context in daily life, with little recall bias and high ecological validity. The DizzyQuest reached the desired response rates and showed different disease profiles between subgroups of patients with different vestibular disorders. This is the first time ESM was used to assess daily symptoms and quality of life in vestibular disorders, showing that it might be a useful tool in this population.

**Electronic supplementary material:**

The online version of this article (10.1007/s00415-020-10092-2) contains supplementary material, which is available to authorized users.

## Introduction

Dizziness, imbalance and vertigo are common complaints, with a prevalence of up to 36% in the outpatient population [1]. A subset of these complaints is caused by vestibular disorders [2]. These vestibular disorders can present with different clinical pictures, varying from acute attacks of vertigo to chronic complaints [3–5]. Patients with a vestibular disorder can also suffer from other problems like depression, increased risk of falling and cognitive impairment, with many socio-economic consequences [6]. To evaluate the symptoms and consequences of vestibular disorders, questionnaires like the Dizziness Handicap Inventory (DHI) [7], the Hospital Anxiety and Depression Scale (HADS) [8], the Short-Form Health Survey (SF-36) [9], Short Falls Efficacy Scale-International (Short FES-I) [10], and the EQ-5D-5L are often used.

However, there are two main drawbacks tied to the use of cross-sectional instruments. First, most of these questionnaires are retrospective, and require recollection and adequate aggregation. This imposes a high risk of recall bias, since some variables might be overestimated or underestimated. Secondly, most of these questionnaires only yield one observation and miss documenting variability in symptomatology or risk patterns in the context of daily life. Cross-sectional instruments are not able to capture dynamic components (e.g., fluctuating symptoms) and the influence of context (e.g., environmental or psychological factors, etc.), which are relevant indicators for symptom management. Understanding symptoms in the normal flow of daily life improves the ecological validity: it better reflects the real life response of symptoms to real life challenges [11–13]. Especially in vestibular disorders which can have a very fluctuating clinical presentation with vertigo attacks, questionnaires prone to recall bias and lower ecological validity, are suboptimal to study the exact frequency of attacks, their possible triggers and the quality of life in vestibular disorders.

In the past, the Experience Sampling Method (ESM) was developed to overcome recall bias and to increase the ecological validity of measurements of health outcomes. Historically, paper and pencil procedures were burdensome. Recently, apps on a smartphone improved data collection logistics. In ESM the information about an individuals’ current state and functioning, as well as circumstances and context, is documented by self-report in real-time using a random time sampling procedure to repeatedly fill in questionnaires for a longer period of time. The questionnaires have to be filled out multiple times a day, at most 10 times. On these moments, a notification beep alerts the subject to fill out an assessment form. A questionnaire can also be filled out by the subject in case of a certain trigger or event [11]. ESM not only reduces the risk of recall bias, it also makes it possible to obtain information about the variability of attacks over time [13]. Furthermore, the existence and influence of environmental, social and psychological factors can be investigated at the same time [14]. Momentary assessment has already been successfully used in studies on other diseases, e.g., tinnitus [15], psychological disorders [16–18] and Parkinson’s disease [19]. However, there is only scarce evidence in vestibular disorders, using techniques that to a certain extent resemble ESM [14].

Since patients often bring their own (non-standardized) “vestibular diary” to the clinic and currently many new therapeutic strategies are being investigated [20–40], standardized monitoring of patients using ESM improves clinical work and research, complementary to existing questionnaires. Therefore, the Dizzynet network [41, 42] developed a new app-based diary for vestibular disorders: the DizzyQuest. Main objective of this study was to describe the first version of the DizzyQuest, to evaluate response rates, and to provide examples of DizzyQuest outcome measures which can be used in future research, like the variability of neuro-otological symptoms over time using the ESM method, and the relationships between symptoms and their psychosocial context.

## Methods

### Study participants

Patients with various vestibular disorders were recruited at the vestibular department of Maastricht University Medical Center, Apeldoorn Dizziness Center, Haga Hospital and the ‘Hoormij’ foundation, all in the Netherlands. Inclusion criteria comprised: (1) age 18 years or older; (2) diagnosed with a vestibular disorder by an otorhinolaryngologist with an expertise in vestibular disorders, according to the Barany Society diagnostic criteria [43–46] (if applicable); (3) good understanding of the Dutch language (reading and listening); (4) in possession of a smartphone or tablet; (5) being capable to install and use the DizzyQuest (see below for explanation of the application). Patients were excluded if they did not feel comfortable to answer questions from the DizzyQuest, for example, concerning their psychological status.

### The DizzyQuest

The DizzyQuest is an app-based electronic diary that is implemented on the “UM ESM” (v2.04) platform. This is an experimental version of the PsyMate™ app (http://www.psymate.eu) used for research at Maastricht University. The app allows implementation of ESM based protocols with multiple questionnaires and sampling schemes [47]. It is available as a download for smartphones running on iOS and Android. The DizzyQuest consists of four questionnaires. The “within-day-questionnaire” assesses the current situation at semi-random intervals throughout the day in the tradition of the ESM method for 10 times a day. The other three questionnaires are retrospective, in principle reflecting an aggregated situation of the previous night (morning questionnaire), or previous day (evening questionnaire). The attack questionnaire is event based and filled in after the occurrence of an attack of vertigo or dizziness (Table 1). It can be completed at any time after an attack, even after more than 1 day (Online Resource 1). Each questionnaire has its own specific set of questions, although considerable overlap exists between some of them (e.g. the within-day-questionnaire and the evening-questionnaire, see Online Resource 1 for the translated version). All four questionnaires combined, not only measure vestibular symptoms, but also emotion, cognition, context and stressful events. All questions about symptoms use the seven-point Likert scale, ranging from 1 (not at all) to 7 (very). It should be noted that the questionnaires were provided in Dutch. In this language, “dizziness” and “vertigo” are often used interchangeably in contrast to the English language, in which “dizziness” and “vertigo” are considered two different entities [48].

**Table 1:** Questionnaires used in the DizzyQuest

Questionnaire	Focus	Nature	Frequency in this study
Morning (7 questions)	Quality of sleep past night	Retrospective	Daily in first week
Within-day (23 questions)	Real-time symptoms in daily life context	Momentary (= ESM)	Ten times a day at semi-random moments in the first week
Evening (27 questions)	Symptoms of the past day	Retrospective	Daily during 4 weeks
Attack (5 questions)	Symptoms of attack	Retrospective	After an attack of vertigo or dizziness

These different strategies (momentary versus retrospective) and different questionnaires, provide the opportunity to measure multiple aspects of neuro-otological symptoms and their psychosocial context. Table 2 provides possible outcome measures that can be obtained by the DizzyQuest on an individual level.

**Table 2:** Possible outcome measures of the DizzyQuest (on individual level)

Outcome measure	Facilitating questionnaire
Behaviour of symptoms during the day	Within-day
Behaviour of symptoms between days	Morning
	Within-day
	Evening
Appearance of neuro-otological symptoms in relation to psychosocial context	Morning
	Within-day
	Evening
Frequency and nature of attack of vertigo or dizziness	Attack
	Evening

### Study design

Before start of the DizzyQuest study, patients were contacted by two of the authors (SvdW, AE) and instructed on how to use the app. An online video was used for instructing the patients how to use the DizzyQuest (Online Resource 2). After that, the DizzyQuest was used for four sequential weeks. In this study, not all questionnaires were applied in the same frequency and for the same duration (Table 1). In the first week, each day contained one morning-questionnaire, 10 within-day-questionnaires and one evening-questionnaire. Subjects received the within-day-questionnaires 10 times a day, between 07:30 a.m. and 22:30 p.m., at semi-random moments (a day consisted of ten 90-min blocks and at a random moment in each block, a questionnaire was sent to the patient). The morning-, day- and evening-questionnaires were activated at 04:00 a.m. and 07:30 p.m. respectively, with a reminder on the smartphone or tablet at 9:00 a.m. and 8:00 p.m. respectively. They could be answered for a period of 8 and 8.5 h, respectively. The within-day-questionnaires were activated after a signalling beep/notification on the device. The subject was able to fill in the questionnaire within a time window of 15 min. When the opportunity to fill in the questionnaire closed, the questionnaires disappeared from the home screen and data were archived as “negative response”. The attack-questionnaire could be used at any given moment, as many times a day as needed. Subjects were instructed on the first day and debriefed on day 28. After the first week, only the evening- and attack-questionnaires remained. Therefore, in the last 3 weeks, patients only had to fill in one questionnaire a day (the evening-questionnaire), and optionally the attack-questionnaire in case of an attack of vertigo or dizziness, to reduce the burden for subjects.

### Patient monitoring

The ESM UM Reporting Tool provides the opportunity for online monitoring of results. It was used to remotely monitor compliance of patients by two of the authors (SvdW, AE). In case a patient did not start the DizzyQuest at the predetermined day, or when sequentially multiple questionnaires were not answered, patients were contacted by phone or e-mail to provide any help if necessary (e.g., technical help).

### Data analysis

Patients that started in the first week and continued the use of the DizzyQuest up to and including the fourth week, were included in the analysis. No minimum threshold of response rates was required. DizzyQuest data were extracted from the ESM UM database and transferred to IBM SPSS Statistics Version 25 (IBM Corporation, New York, United States of America). Data from the first day (briefing) was incomplete and skipped from the analysis, leaving only within-day-questionnaire data from day two until seven. The morning questionnaire was available from day two until eight, resulting in 7 days of data to be used for analysis. The evening- and attack-questionnaires were available for the whole 28 days period. Descriptive statistics were used. Furthermore, a Mann–Whitney U test, Fisher’s Exact test and a Fisher-Freeman-Halton exact test of independence were used to calculate differences in age, gender and diagnosis respectively, between the study group and drop-out group. The average response rate of each patient for each questionnaire was calculated by dividing the amount of fully completed questionnaires by the number of scheduled questionnaires. Only fully completed questionnaires were considered as valid. It was hypothesized that not all administered questionnaires would be completed. A desired response rate of > 50% was used, since reported response rates in current literature about momentary assessments are often higher than 50% [49, 50]. To calculate whether response rates of the DizzyQuest questionnaires were significantly different from the > 50% desired response rate [47, 51], a one-sample *T* test was performed.

Regarding presence of neuro-otological symptoms, they were considered “reported” and present, when they were selected in the question: ‘Now I suffer from’ (Online Resource 1). The reported frequency (%) of neuro-otological symptoms obtained with the within-day questionnaire, was first calculated for each symptom per patient. This was done by dividing the amount of times a specific symptom was reported, by the amount of times the individual patient completed the within-day questionnaire. This resulted in a patient-specific percentage. This patient-specific percentage was used to determine the mean reported frequency (%) of all patients, divided in three subgroups of vestibular disorders: Menière’s disease (MD), bilateral vestibulopathy (BV), and other vestibular disorders.

Marginal logistic regression analysis based on the generalized estimated equations analysis (GEE) method for binary outcomes was used to test significant differences in reported symptoms between subgroups of vestibular disorders and the outcomes were Bonferroni corrected. An exchangeable covariance structure was applied, since time interval between within-day questionnaires was not constant.

To investigate the effect of neuro-otological symptoms on questions regarding positive affect and negative affect, linear mixed-effects regression analysis was performed. The independent variables in the models were one of the neuro-otological symptoms (“imbalance”, “tinnitus” and “visual problems when moving”). The score on one of the questions regarding positive affect (“I generally feel well at the moment”) and negative affect (“I’m worrying about things”) was the dependent variable. In case of a significant interaction was found between the diagnosis group and the neuro-otological symptom, the analysis was performed for each diagnosis group separately.

To investigate differences between the three subgroups regarding amount of completed attack-questionnaires, the mean number of completed attack-questionnaires was first calculated per day for each patient by [amount of attack questionnaires completed by a specific patient/28 days]. Subsequently, a Kruskal–Wallis test was used to test for systematic differences between the three groups. In case of a significant Kruskal–Wallis test, post hoc analyses included three pairwise Bonferroni corrected Mann–Whitney U tests. For all performed statistic tests, *p*-values below 0.05 were considered statistically significant.

### Ethical considerations

This study was in accordance with the legislation and ethical standards on human experimentation in the Netherlands and in accordance with the Declaration of Helsinki (amended version 2013). The medical ethical committee of Maastricht UMC+ approved this study (2018-0809) and written informed consent was obtained from all patients, prior to participation.

## Results

### Patient characteristics and drop-out

Sixty-three patients were included in this study. Twelve patients dropped out of the study for a variety of reasons (e.g., technical problems or personal circumstances) as described in Table 3. Fifty-one patients completed the 4-week study period. This group consisted mostly of females (*n* = 33) and had a mean age of 56 years old (range 27–80 years). Etiologies comprised Menière’s disease (*n* = 19), vestibular migraine (*n* = 3), vestibulopathy (*n* = 1 unilateral, *n* = 23 bilateral) and overlap syndrome between Menière’s disease and vestibular migraine (*n* = 5). There were no significant differences between the patients who completed the study period and the patients who dropped out, regarding age (*p* = 0.475), gender (*p* = 0.309) and vestibular diagnosis (*p* = 0.367).

**Table 3:** Characteristics of patients who dropped out during this study

Patient number	Diagnosis	Gender	Age (years)	Reason for drop-out
1	VM/PPPD	Female	63	Prioritized treatment for another medical disorder
2	MD	Female	43	Could not combine the application with work
3	MD	Female	37	Changed her mind regarding participation after inclusion in study
4	BV	Female	44	Prioritized treatment for another medical disorder
5	PPPD	Female	51	Technical problems: did not receive evening questionnaires after using another device
6	MD	Female	59	Technical problems: incorrect login
7	MD	Female	52	Technical problems: incorrect login
8	MD	Male	59	Frequency of within-day questionnaires was too high to combine participation in this study with personal life
9	DFNA9	Male	70	Technical problems: dissatisfied about the application
10	MD	Female	64	Personal circumstances
11	UVP	Female	61	Technical problems: application did not work properly
12	MD	Female	44	Death of family member

*VM* vestibular migraine, *PPPD* persistent postural perceptual dizziness, *MD* Menière’s disease, *UVP* unilateral vestibulopathy, *DFNA9* genetic type of cochleovestibular dysfunction, leading to deafness and bilateral vestibulopathy

### Response rates

**Fig. 1:**
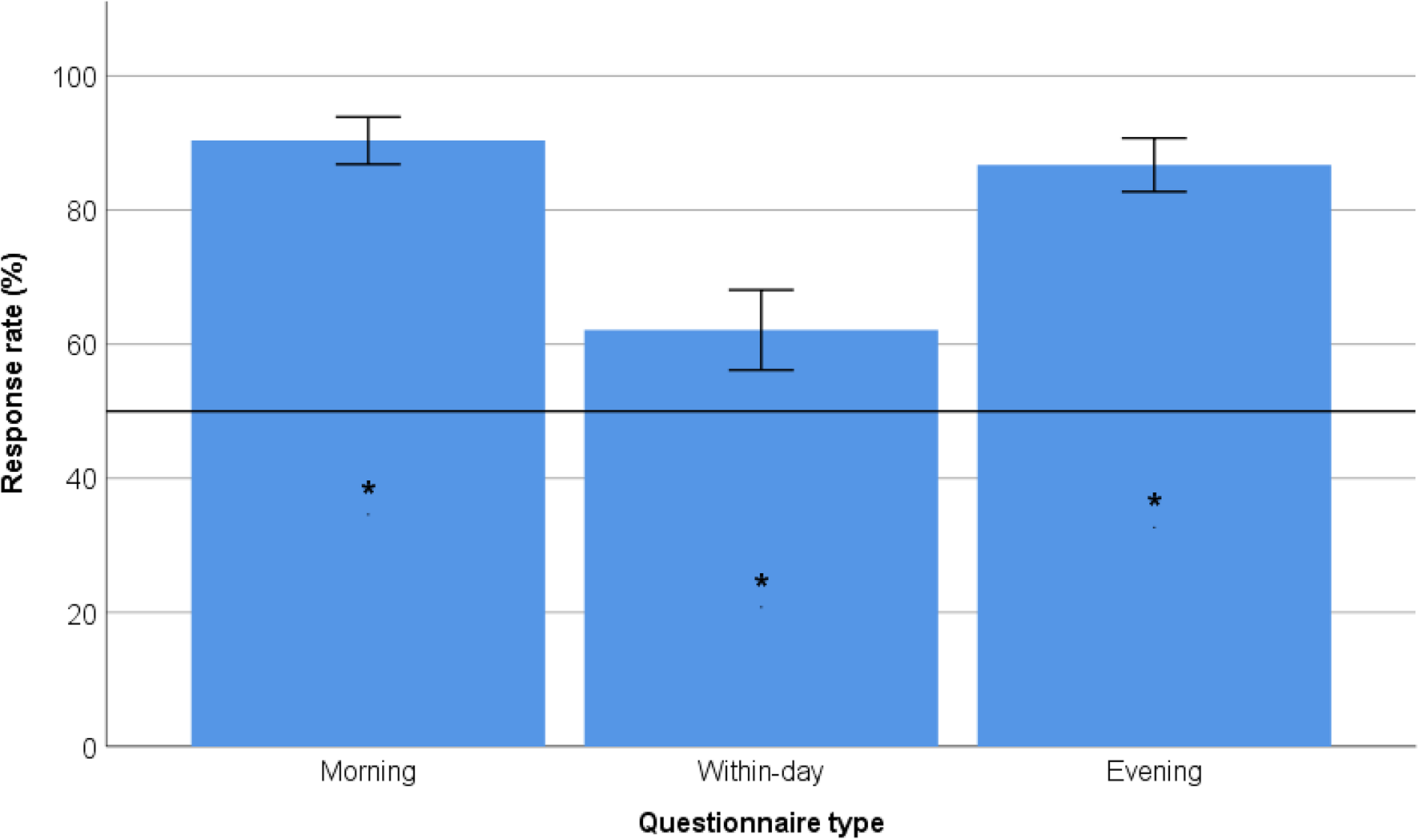
The average response rates of fully completed questionnaires of the DizzyQuest. Error bars indicate 95% confidence intervals of average response rates of all participants on the specific questionnaire. The black horizontal bar indicates the desired response rate of > 50%. Asterisks indicate a statistically significant difference in response rate from the desired > 50% response rate

Figure 1 presents the average response rates of fully completed questionnaires of the DizzyQuest. The response rates were 90% for the morning-questionnaire, 62% for the within-day-questionnaire and 87% for the evening-questionnaire. The response rates of the morning, day and evening questionnaires were significantly different from the desired a priori defined response rate of > 50% (*p* < 0.001) respectively.

### Examples of outcome measures of the DizzyQuest on an individual level

**Fig. 2:**
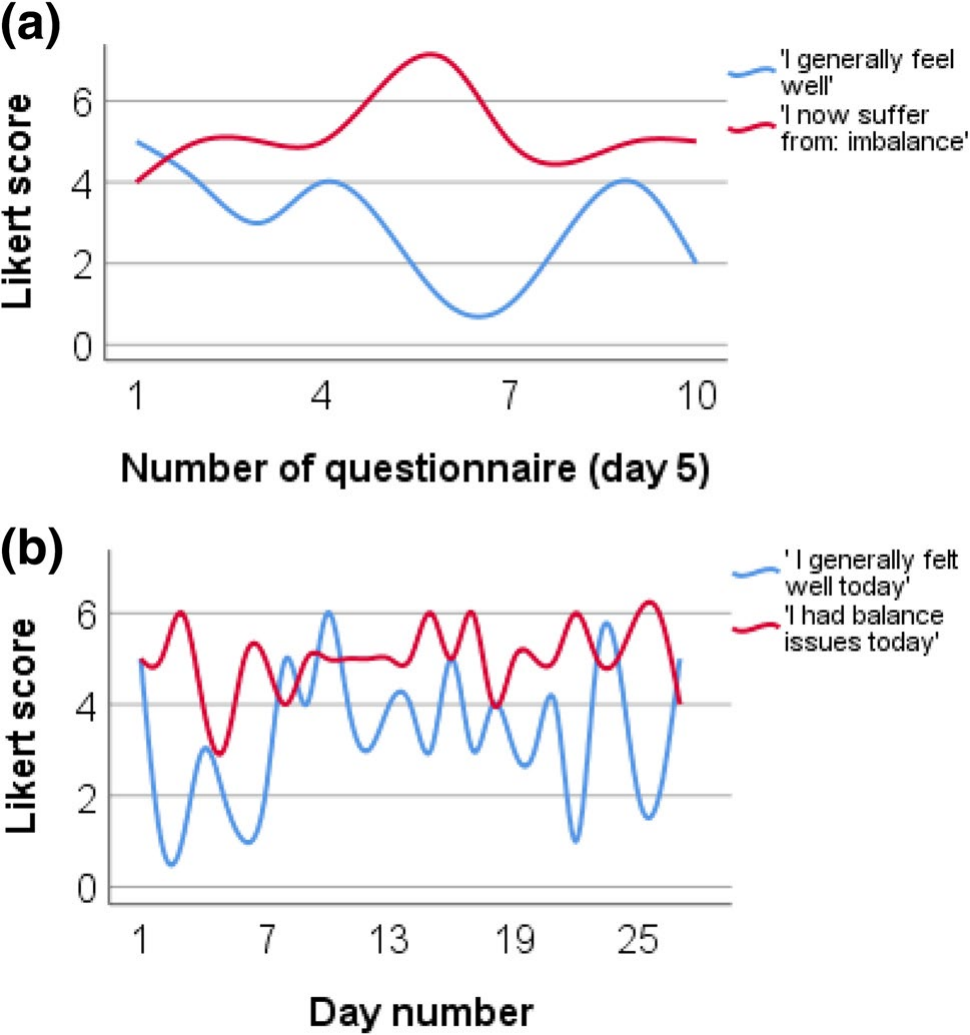
**a**, **b** Examples of DizzyQuest results of a patient with Menière’s disease regarding imbalance (red) and general well-being (blue) during one specific day and 1 month, as a representative sample of results obtained in this study. Likert scores on the y-axes vary from 1 (not at all) to 7 (very). **a** Illustrates the results of the 10 within-day-questionnaires that were administered on day five of the study. **b** Illustrates the results of the evening questionnaires obtained during 28 consecutive days

**Fig. 3:**
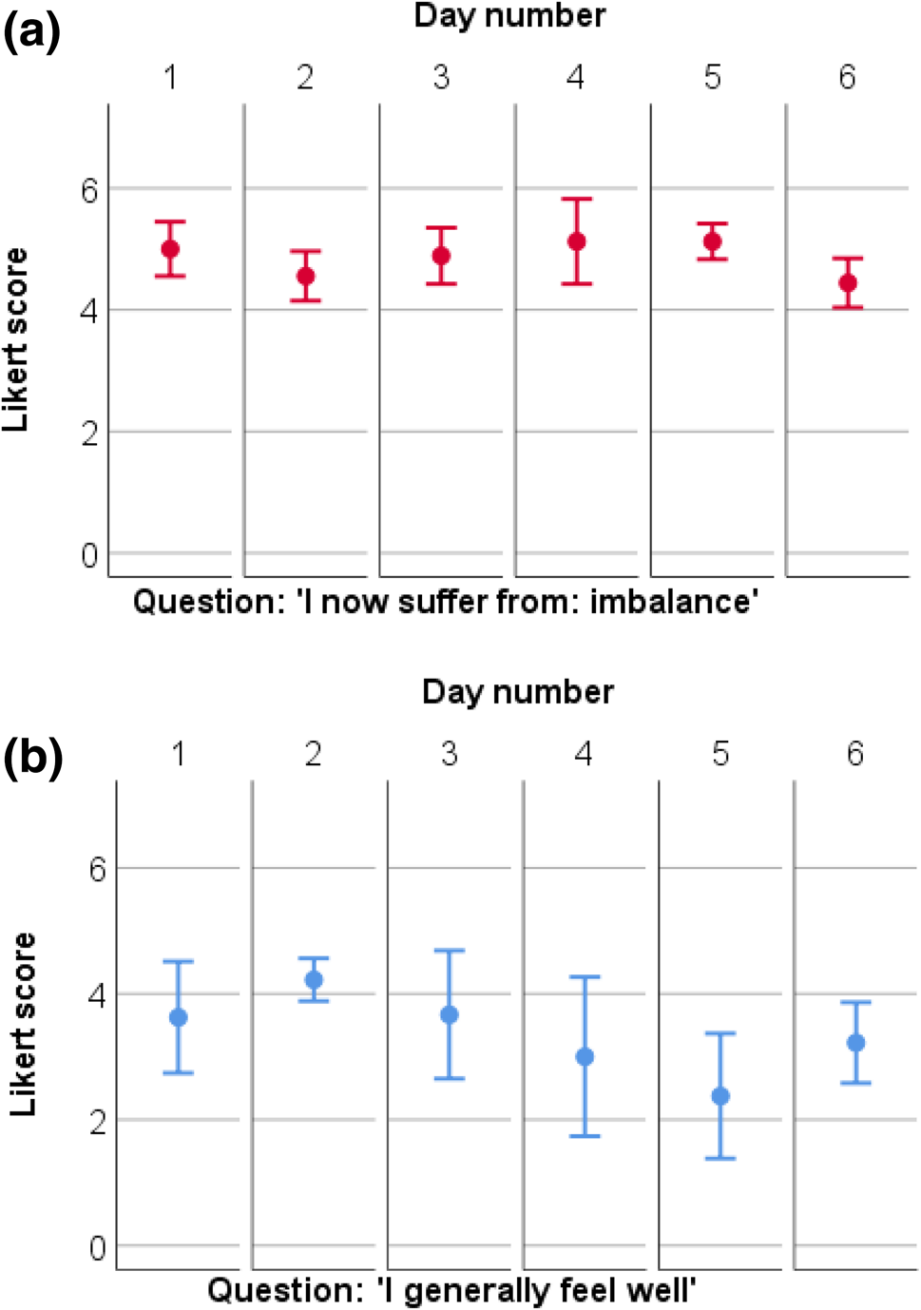
**a**, **b** Average daily scores of imbalance (red, **a**) and general well-being (blue, **b**) obtained with the within-day-questionnaires that were administered 10 times a day during six consecutive days, in the same patient with Menière’s disease. Dots represent the average scores of all completed within-day-questionnaires during a specific day. Error-bars show the 95% confidence intervals

The DizzyQuest enables the opportunity to give additional insights in fluctuation of symptoms during a specific time period (e.g., day or week), and to investigate the appearance of symptoms in relation to psychosocial context (Table 2). Figure 2a, b present examples of the association of imbalance and general well-being during a day (Fig. 2a) and a month (Fig. 2b) in a patient with Menière’s disease, as a representative sample of results obtained in this study. Figure 3 presents the average scores of all 10 administered within-day-questionnaires during six consecutive days regarding imbalance and general well-being in the same patient. These figures show that with the DizzyQuest, it is possible to calculate correlations between the appearance of symptoms and their context, on an individual and group level (see below “Appearance of neuro-otological symptoms in relation to psychosocial context”).

### Examples of outcome measures of the DizzyQuest on group level

#### Frequency of reported symptoms in the within-day-questionnaire (ESM method)

**Fig. 4:**
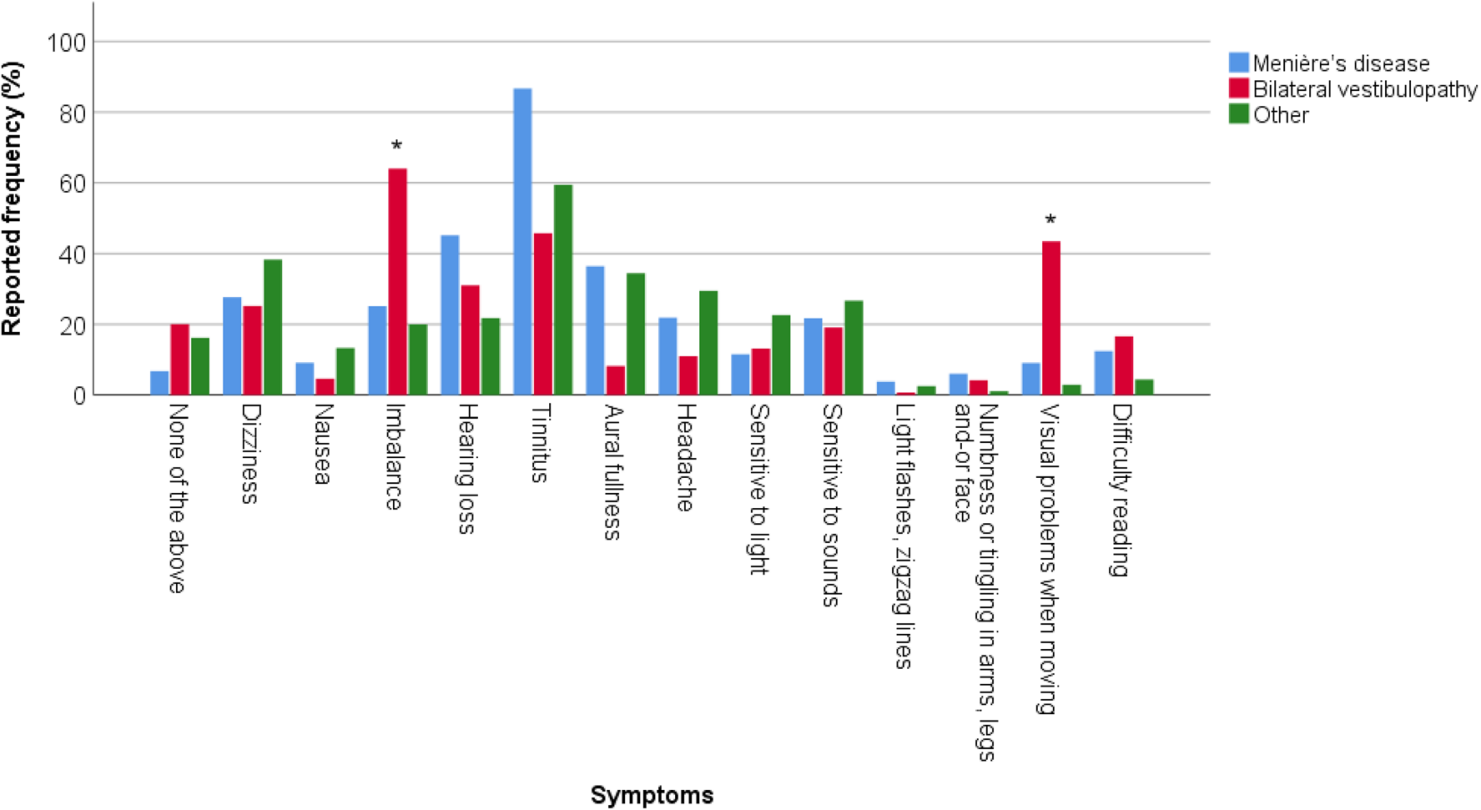
Mean reported frequency (%) of neuro-otological symptoms, obtained with the within-day questionnaire, during six consecutive days in 51 patients, divided in three subgroups of vestibular disorders. Asterisks above a subgroup bar indicate a statistically significant difference in reported frequency of a symptom in that specific subgroup, compared to the other two

In total, 51 patients reported 5829 neuro-otological symptoms in the within-day-questionnaire. Figure 4 demonstrates the mean reported frequency (%) of neuro-otological symptoms, obtained with the within-day questionnaire, during six consecutive days in 51 patients. These are graphically displayed in three subgroups by diagnosis: Menière’s disease (*n* = 19), bilateral vestibulopathy *(n* = 23), and other vestibular disorders (*n* = 9). Mostly reported symptoms were tinnitus and imbalance, while light flashes were least reported. Furthermore, variability can be observed between type of symptoms and subgroups of vestibular disorders. Patients with bilateral vestibulopathy reported imbalance significantly more often than patients with Menière’s disease (*p* = 0.003, OR = 5.279, 95% CI 1.917; 14.533) and patients with other vestibular diagnoses (*p* = 0.012, OR = 0.142, 95% CI 0.037; 0.540). Patients with bilateral vestibulopathy reported also significantly more often visual problems when moving compared to patients with Menière’s disease (*p* = 0.033, OR = 7.735, 95% CI 1.595; 37.524) and patients with other vestibular diagnoses (*p* < 0.001, OR = 0.038, 95% CI 0.012; 0.126).

#### Appearance of neuro-otological symptoms in relation to psychosocial context

The appearance of three neuro-otological symptoms (imbalance, tinnitus and visual problems while moving), and their association with questions about positive affect (“I generally feel well at the moment”) and negative affect (“I’m worrying about things”) is presented in Table 4. Linear mixed-model regression analysis showed that the appearance of imbalance, tinnitus and visual problems while moving, were nearly always significantly associated with a decrease in “I generally feel well at the moment” for all subgroups of vestibular disorders (except visual problems when moving in patients with Menière’s disease). Regarding the question “I’m worrying about things”, the presence of imbalance was associated with a lower score.

**Table 4:** Results of the mixed-effects linear regression analyses regarding the appearance of three neuro-otological symptoms and two questions about positive affect and negative affect, calculated for three subgroups of vestibular disorders

Symptoms	Questions regarding positive affect and negative affect							
	I generally feel well at the moment				I’m worrying about things			
	Estimate	95% CI		*p*-value	Estimate	95% CI		*p*-value
		Lower	Upper			Lower	Upper	
Imbalance = no	− 0.466^a^	− 0.697	− 0.234	< 0.001*	0.193^a^	0.044	0.341	0.014*
Tinnitus = no	− 0.413^a^	− 0.670	− 0.155	0.003*	0.177^a^	− 0.057	0.411	0.132
Visual problems when moving = no	BV: − 0.234	− 0.440	0.029	0.025*	0.12^a^	− 0.046	0.290	0.156
	Other: − 1.163	− 1.877	− 0.450	0.001*				
	MD: − 0.147	− 0.624	0.331	0.547				

*BV* bilateral vestibulopathy, *MD* Menière’s disease

*p < 0.05

^a^Applicable to all subgroups

#### Frequency and nature of attacks of vertigo or dizziness

The attack-questionnaire was used 159 times to indicate an attack of vertigo or dizziness. The median of number of reported vertigo attacks in the attack questionnaire per day was 0.071 for patients with Menière’s disease, 0.036 for patients with bilateral vestibulopathy and 0.143 for patients with other vestibular disorders. Patients in the subgroup “other vestibular disorders” reported significantly more often a vertigo attack than patients in the subgroup “bilateral vestibulopathy” (*p* = 0.033).

On an individual level, it was observed that the attack- and evening-questionnaires showed different results regarding the reported frequency and nature (e.g., triggers, duration) of attacks of vertigo or dizziness. This interesting finding was outside the scope of this article, since it mainly results from the differences between reporting strategies: event sampling (attack-questionnaire) and time sampling (evening-questionnaire). This will therefore be discussed separately [52].

## Discussion

The objective of this study was to introduce the first version of the DizzyQuest, to evaluate its response rates, and to provide examples of DizzyQuest outcome measures which can be used in future research. Response rates to all investigated questionnaires of the DizzyQuest were significantly higher than the desired response rate of > 50%. The DizzyQuest is able to capture a variety of symptoms, including significant variability of symptomatology within and between participants and between subgroups of vestibular disorders. Furthermore, it was demonstrated that the Dizzyquest facilitates investigation of significant relations between symptoms and their psychosocial context. This is the first time that ESM has been used to assess symptoms in a population with vestibular disorders.

Main advantage of the DizzyQuest is the fact that it dynamically captures symptoms and their psychosocial context, by administering questionnaires multiple times to the same patient during patients’ daily life. Therefore, it allows specific insights in the course of symptoms over time, and how they correlate with psychosocial factors. The DizzyQuest measures “in the moment” (within-day-questionnaire) or with limited retrospection (morning-, evening- and attack-questionnaire), herewith decreasing recall bias and improving reliability. Since it is used in daily life, the ecological validity might be improved compared to many currently available questionnaires which are administered once a week at most. These aspects will likely be very valuable when investigating the effect of therapies for vestibular disorders, especially in cases of fluctuating symptoms like in Menière’s disease [40, 53]. In the future, it could open doors to ecological momentary interventions, which take place in real time and in real world [54]. The repetitive measurement of the DizzyQuest also provides much larger numbers of data about the same topics, in and between patients. This allows for higher statistical accuracy in a smaller number of patients, and provides the opportunity of patients serving as their own control condition during intervention studies (e.g., *n* = 1 study [55]). Moreover, symptom occurrence and worsening can be understood, since ESM allows the study of the mutual interaction of symptoms. Taking these considerations into account, the DizzyQuest could be used as a (complementary) tool for investigating symptoms and quality of life in patients with vestibular disorders.

Average response rates of the morning-questionnaire were the highest, while those of the within-day-questionnaire were the lowest. Additionally, the drop-out rate was 19% of patients. Fortunately, no patients dropped out due to any experienced psychological burden related to DizzyQuest use. Mostly reported reasons for drop-out involved technical problems and personal circumstances (e.g., combining the DizzyQuest with daily life activities). Regarding technical problems, these might be addressed by more strict monitoring of the patients in future trials, and to provide technical help if necessary. However, personal circumstances as a reason for drop-out, imply that responding to random signals is demanding in a practical way. Subjects have only 15 min to respond. Especially the within-day-questionnaire could be perceived as a burden, according to patients’ feedback. After all, the within-day-questionnaire was administered 10 times a day in the first week. By providing questions 10 times a day, it was aimed to reach at least five completed within-day-questionnaires per day. Therefore, a lower response rate was expected compared to the morning and evening DizzyQuest questionnaires. Although the desired response rate was reached, the average response rate on the within-day-questionnaire in the patients that completed the whole study period, was slightly lower than in similar research across different domains [56, 57]. It is therefore strongly advised, especially when using the demanding within-day-questionnaire, to provide patients with proper guidance during the study period. This might increase adherence [58]. Another complementary strategy would be to lower the within-day-questionnaire frequency, however, at the risk of getting too little data.

A variety of symptoms was reported within and between patients. It should be noted that these symptoms reflect the study population, and not the patients with vestibular disorders in general. For example, tinnitus was the most reported symptom, probably reflecting the overrepresentation of Menière’s disease patients in this study population. Nevertheless, it has been shown that the DizzyQuest captured different profiles of symptomatology between subgroups of vestibular disorders. For example, imbalance and visual problems while moving (referring to oscillopsia) were significantly more reported in patients with bilateral vestibulopathy. These findings were expected, since they (partially) reflect the diagnostic criteria established for this disorder [46]. In addition, patients with bilateral vestibulopathy reported on average the lowest amount of vertigo attacks. Taking these results into account, it can be concluded that the DizzyQuest is able to measure different profiles of symptomatology between patients with different vestibular disorders.

Furthermore, the DizzyQuest facilitated the investigation of the relationship between neuro-otological symptoms and their psychosocial context. It was demonstrated that psychosocial well-being can be significantly negatively influenced by neuro-otological symptoms like imbalance, tinnitus and oscillopsia. This is congruent with previous literature in which it was shown that patients with vertigo or dizziness are more prone to have psychiatric co-morbidity and in these cases more often report depression, anxiety and vertigo-related handicap [59]. The DizzyQuest might therefore be used as a complementary tool to further explore the interface between neuro-otological symptoms and their psychosocial context in future research.

### Limitations

A subset of the study population was loss-to-follow-up. This implies that results are only a reflection of the patients that completed the whole 4-week study period. Whether this has induced any selection bias, cannot be ruled out. Furthermore, completing the questionnaires might take some time, especially in the beginning. However, bothersome time consumption was not measured in this study. After familiarization with the questionnaires, time to complete each questionnaire dropped to on average 2 min, according to the patients’ feedback.

In this first article about the DizzyQuest, it would have been preferred to also report on its content and construct validity. However, since the development of the DizzyQuest was a very long and thorough process according to the COSMIN guidelines [60], it was decided to report this separately in an article fully devoted to the validity of the DizzyQuest (manuscript in preparation).

### Future steps to DizzyQuest

To fully capture symptoms of patients and their psychosocial context, focus groups were recently conducted, in which the questionnaires were discussed in a standardized way [56, 61]. Patients and doctors got the opportunity to include, exclude and rephrase questionnaire items. These results will be implemented in the updated version of the DizzyQuest. Furthermore, the DizzyQuest should also be validated, including comparison to currently used questionnaires like the DHI and HADS (concurrent validity), and it should be translated into multiple languages. Preliminary observations showed in some cases discrepancies between reported symptoms in the within-day-questionnaire and the evening-questionnaire (data not shown). This is probably due to the different nature of the questionnaires (momentary versus retrospective design) and their timing (multiple times a day versus only one time during the evening).

Moreover, recent technological innovations resulted in portable hearing tests [62], video-oculography goggles for home use [63] and wearable movement sensors [64]. Simultaneous data-acquisition of these devices in combination with the DizzyQuest during daily life, might result in clinically useful insights regarding the correlation between subjective symptoms and objective measurements in patients with vestibular disorders. Finally, it should be noted that the frequency of administering the DizzyQuest questionnaires is not fixed. For example, in this study the within-day-questionnaire was administered 10 times a day for 1 week. However, this could be changed to e.g., 6 times a day for 2 weeks. Currently, two versions of the DizzyQuest are being developed for use in the near future: an updated extensive version for research including all questionnaires mentioned in this study, and a short DizzyQuest including only the evening- and attack-questionnaire. This latter should be able to serve as a standardized vestibular diary with relatively little retrospectivity, but without the burden of the within-day-questionnaire. However, it should be noted that not using momentary assessments might decrease the sensitivity and the ability to investigate symptom appearance in relation to psychosocial context [65].

## Conclusion

The DizzyQuest is able to capture vestibular symptoms within their psychosocial context in daily life, with little recall bias and high ecological validity. The DizzyQuest reached the desired response rates and showed different disease profiles between subgroups of patients with different vestibular disorders. This is the first time ESM was used to assess daily symptoms and quality of life in vestibular disorders, showing that it might be a useful tool in this population.

## Electronic supplementary material

Below is the link to the electronic supplementary material.

Supplementary material 1 (PDF 248 kb)

Supplementary material 2 (M4V 434046 kb)
