# Supplementary material for: Introducing the DizzyQuest: an app-based diary for vestibular disorders
Source: J Neurol. 2020 Jul 25;267(Suppl 1):3–14. doi: 10.1007/s00415-020-10092-2 (PMC7718207; doi:10.1007/s00415-020-10092-2)
Supplement: Supplementary file 1 — Supplementary material 1 (PDF 248 kb) [file 415_2020_10092_MOESM1_ESM.pdf]

Introducing the DizzyQuest: An app-based diary for vestibular disorders

Journal of Neurology

E.C. Martin[1], C. Leue[2], P. Delespaul[2], F. Peeters[3], A.M.L. Janssen[4,5], R. Lousberg[2], A. Erdkamp[6], S. van de Weijer[6], J. Widdershoven[1], H. Blom[7], T. Bruintjes[8], A. Zwergal[9], E. Grill[10], N. Guinand[11], A. Perez-Fornos[11], M.R. van de Berg[1], J. Stultiens[1], H. Kingma[1, 12], R. van de Berg[1, 12]

Corresponding author: R. van de Berg, raymond.vande.berg@mumc.nl

[1] Division of Balance Disorders, Department of Otorhinolaryngology and Head and Neck Surgery, Maastricht University Medical Center+, Maastricht, Netherlands

[2] Department of Psychiatry and Neuropsychology, School for Mental Health and Neuroscience, Maastricht University Medical Center+, Maastricht, Netherlands

[3] Department of Clinical Psychological Science, Faculty of Psychology and Neuroscience, Maastricht University

[4] Department of ENT/Audiology, School for Mental Health and Neuroscience (MHENS), Maastricht University Medical Centre, Maastricht, Netherlands.

[5] Department of Methodology and Statistics, Care and Public Health Research Institute (CAPHRI), Maastricht University, Maastricht, Netherlands,

[6] mHealth, Maastricht University Medical Center+, Maastricht, Netherlands

[7] Department of ENT, HagaZiekenhuis, The Hague.

[8] Apeldoorns duizeligheidscentrum, Gelre ziekenhuizen, Apeldoorn

[9] Department of Neurology, Ludwig-Maximilians-University of Munich

[10] Department of Medical Informatics, Ludwig-Maximilians-University of Munich

[11] Service of Otorhinolaryngology Head and Neck Surgery, Department of Clinical Neurosciences, Geneva University Hospitals, Geneva, Switzerland.

[12] Faculty of Physics, Tomsk State Research University, Tomsk, Russia

1.1 Morning Questionnaire

Question	Scoring system	Answer options
How long did it take before I fell asleep last night	Multiple choice option	1. 0-5 min<br>2. 5-15 min<br>3. 15-30 min<br>4. 30-45 min<br>5. 45m-1 hour<br>6. 1-2 hour<br>7. 2-4 hours<br>8. More than 4 hours
How long did I sleep?	Multiple choice option	1. <3 hours<br>2. 3-5 hours<br>3. 5-8 hours<br>4. >8 hours
How often did I wake up last night	Multiple choice option	1. 0<br>2. 1<br>3. 2<br>4. 3<br>5. 4<br>6. 5<br>7. More than 5 times
How long did I lie awake this morning before getting up	Multiple choice option	1. 0-5min<br>2. 5-15min<br>3. 15-30min<br>4. 30-45min<br>5. 45m-1hour<br>6. 1-2hours<br>7. 2-4hours<br>8. More than 4 hours
I slept well	Likert scale	1 = Not at all<br>4 = Moderate<br>7 = Very
I feel rested	Likert scale	1 = Not at all<br>4 = Moderate<br>7 = Very
I am looking forward to today	Likert scale	1 = Not at all<br>4 = Moderate<br>7 = Very

1.2 Within-Day Questionnaire

Question	Scoring system	Answer options
I feel relaxed	Likert scale	1 = Not at all<br>4 = Moderate<br>7 = Very

I feel cheerful	Likert scale	1 = Not at all<br>4 = Moderate<br>7 = Very
I feel irritated	Likert scale	1 = Not at all<br>4 = Moderate<br>7 = Very
I feel confident	Likert scale	1 = Not at all<br>4 = Moderate<br>7 = Very
I'm worrying about things	Likert scale	1 = Not at all<br>4 = Moderate<br>7 = Very
I feel anxious	Likert scale	1 = Not at all<br>4 = Moderate<br>7 = Very
I feel tired	Likert scale	1 = Not at all<br>4 = Moderate<br>7 = Very
I generally feel well at the moment	Likert scale	1 = Not at all<br>4 = Moderate<br>7 = Very
I am able to concentrate well	Likert scale	1 = Not at all<br>4 = Moderate<br>7 = Very
I feel in control	Likert scale	1 = Not at all<br>4 = Moderate<br>7 = Very
What am I doing (just before the beep)?	Multiple choice option (maximum 3 options)	1. Resting<br>2. Work, school<br>3. Housekeeping, shopping<br>4. Hygiene<br>5. Eating, drinking<br>6. Active relaxation<br>7. Passive relaxation<br>8. Something else
I feel I'm being active	Likert scale	1 = Not at all<br>4 = Moderate<br>7 = Very
I can do this well	Likert scale	1 = Not at all<br>4 = Moderate<br>7 = Very
I'd rather be doing something else	Likert scale	1 = Not at all<br>4 = Moderate<br>7 = Very
Where am I?	Multiple choice option	1) At home<br>2) At someone's home<br>3) Work, school<br>4) Public place<br>5) Transport<br>6) Somewhere else

Who am I with?	Multiple choice option (maximum 3 options)	1. Partner<br>2. Family resident<br>3. Family non resident<br>4. Friends<br>5. Colleagues<br>6. Acquaintances<br>7. Strangers, others<br>8. Nobody
I like this (social situation)	Likert scale	1 = Not at all<br>4 = Moderate<br>7 = Very
I have an attack at this moment	Multiple choice option	1= yes<br>2 = no
Since the last beep I have had an attack	Multiple choice option	1 = yes<br>2 = no
Since the last beep I have used	Multiple choice option (maximum 7 options)	1. Caffeine<br>2. Nicotine<br>3. Alcohol<br>4. Medication<br>5. Tea<br>6. Chocolate<br>7. Other substance<br>8. Nothing of these
I now suffer from	Multiple choice option (maximum 13 options)	1. Dizziness<br>2. Nausea<br>3. Imbalance<br>4. Hearing loss<br>5. Tinnitus<br>6. Aural fullness<br>7. Headache<br>8. Sensitive to light<br>9. Sensitive to sounds<br>10. Light flashes, zigzag lines<br>11. Numbness or tingling in arms, legs and-or face<br>12. Visual problems when moving<br>13. Difficulty reading<br>14. None of the above
If one of more symptoms are selected, the subject is then asked to indicate the severity of those symptoms individually	Likert scale	1 = Not at all<br>4 = Moderate<br>7 = Very
Think about the most important thing that has happened since the last beep	No answer option	
This event was	Likert scale	-3 = stressful<br>+3 = relaxing
This beep bothered me	Likert scale	1 = Not at all

		4 = Moderate<br>7 = Very

1.3 Evening Questionnaire

Question	Scoring system	Answer options
This was an ordinary day	Likert scale	1 = Not at all<br>4 = Moderate<br>7 = Very
I generally felt well today	Likert scale	1 = Not at all<br>4 = Moderate<br>7 = Very
I generally felt tired today	Likert scale	1 = Not at all<br>4 = Moderate<br>7 = Very
I generally felt relaxed today	Likert scale	1 = Not at all<br>4 = Moderate<br>7 = Very
I generally worried a lot today	Likert scale	1 = Not at all<br>4 = Moderate<br>7 = Very
I generally was able to concentrate well today	Likert scale	1 = Not at all<br>4 = Moderate<br>7 = Very
I felt dizzy today	Likert scale	1 = Not at all<br>4 = Moderate<br>7 = Very
I felt nauseous today	Likert scale	1 = Not at all<br>4 = Moderate<br>7 = Very
I had balance issues today	Likert scale	1 = Not at all<br>4 = Moderate<br>7 = Very
Today I suffered from hearing loss: left	Likert scale	1 = Not at all<br>4 = Moderate<br>7 = Very
Today I suffered from hearing loss: right	Likert scale	1 = Not at all<br>4 = Moderate<br>7 = Very
Today I suffered from tinnitus: left	Likert scale	1 = Not at all<br>4 = Moderate<br>7 = Very
Today I suffered from tinnitus: right	Likert scale	1 = Not at all<br>4 = Moderate<br>7 = Very
Today I suffered from aural fullness	Likert scale	1 = Not at all<br>4 = Moderate<br>7 = Very
Today I suffered from headache	Likert scale	1 = Not at all<br>4 = Moderate

		7 = Very
Today I suffered from light-sensitivity	Likert scale	1 = Not at all<br>4 = Moderate<br>7 = Very
Today I suffered from sound-sensitivity	Likert scale	1 = Not at all<br>4 = Moderate<br>7 = Very
Today I suffered from light flashes and/or zigzag lines	Likert scale	1 = Not at all<br>4 = Moderate<br>7 = Very
Today I suffered from numbness or tingling in arms, legs and/or face	Likert scale	1 = Not at all<br>4 = Moderate<br>7 = Very
Today I suffered from visual problems when moving	Likert scale	1 = Not at all<br>4 = Moderate<br>7 = Very
Today I suffered from difficulties reading	Likert scale	1 = Not at all<br>4 = Moderate<br>7 = Very
How many attacks of dizziness, nausea and/or hearing loss have you had today?	Multiple choice option	- 0<br>- 1<br>- 2<br>- 3 or more
Did the first attack today already start yesterday?	Multiple choice option	- Yes<br>- No
The attacks were provoked by:	Multiple choice option (maximum 5 options)	- Head or body movements<br>- Strong stimuli (visual, sound, busy places)<br>- Stress<br>- Nothing, started spontaneously<br>- Something else:…
If chosen 'Something else:…' What else provoked the attack?	Open answer option (maximum 120 characters)	
I was unable to function properly because of the attacks for:	Multiple choice option	1. <20 minutes<br>2. 20 minutes – 3 hours<br>3. 3 hours – 12 hours<br>4. >12 hours
To what extent were you limited in your activities today?	Likert scale	1 = Not at all<br>4 = Moderate<br>7 = Very
In which type of activities were you limited the most today?	Multiple choice option (maximum 1 option)	1. Work, study<br>2. Household<br>3. Social<br>4. Travelling, on the way<br>5. Hobbies<br>6. Sports<br>7. Sleeping

1.4 Attack Questionnaire

**Question**	**Scoring system**	**Answer options**
This attack has stopped since	Multiple choice option	1. Happening now<br>2. <20 minutes<br>3. 20 minutes – 3 hours<br>4. 3 hours -12 hours<br>5. >12 hours<br>6. Yesterday or earlier
During this attack I suffered from	Multiple choice option (maximum 13 options)	1. Dizziness<br>2. Nausea<br>3. Imbalance<br>4. Hearing loss<br>5. Tinnitus<br>6. Aural fullness<br>7. Headache<br>8. Sensitive to light<br>9. Sensitive to sounds<br>10. Light flashes, zigzag lines<br>11. Numbness or tingling in arms, legs and-or face<br>12. Visual problems when moving<br>13. Difficulty reading
This attack was provoked by	Multiple choice option (maximum 5 options)	1. Arose automatically<br>2. Head or body movements<br>3. Strong stimuli (visual, sound, busy places)<br>4. Stress<br>5. Something else:…
If chosen 'Something else:…' What other reason was there for the attack?	Open answer option (Maximum 60 characters)	
How severe was this attack?	Likert scale	1 = Very mild<br>7 = Very severe
This attack prevented me from functioning normally for	Multiple choice option	1. <20 minutes<br>2. 20 minutes – 3 hours<br>3. 3 hours – 12 hours<br>4. 12 hours – 1 day<br>5. 1 to 3 days<br>6. >3 days
